# Setting up and relaxation of public health social and physical distancing measures for COVID-19: a rapid review

**DOI:** 10.11604/pamj.supp.2020.35.2.23463

**Published:** 2020-06-12

**Authors:** Jill Ryan, Joseph Okeibunor, Ambrose Talisuna, Charles Shey Wiysonge

**Affiliations:** 1 Cochrane South Africa, South African Medical Research Council, Cape Town, South Africa; 2 World Health Organization Regional Office for Africa, Brazzaville, Congo; 3 School of Public Health and Family Medicine, University of Cape Town, Cape Town, South Africa; 4 Department of Global Health, Stellenbosch University, Cape Town, South Africa

**Keywords:** COVID-19, physical distancing, social distancing, lockdown, setting up, relaxation, exit strategy

## Abstract

**Introduction:**

Physical and social distancing refer to purposeful reduction of close contact between people, such as school closures and workplace closures. These measures are useful in containing coronavirus disease 2019 (COVID-19) but have negative effects on social structures and the economy. There is thus a need for optimal timing on when to setup and relax them. We examined the evidence regarding the initiation and lifting of these public health measures.

**Methods:**

We searched for eligible studies in PubMed, Scopus, and Google Scholar in April 2020, and conducted a qualitative synthesis of the study findings.

**Results:**

We searched for eligible studies in PubMed, Scopus, and Google Scholar in April 2020, and conducted a qualitative synthesis of the study findings. The electronic searches yielded 2503 records, from which we included 10 observational and mathematical modeling studies. These studies used data from one or multiple countries on COVID-19 (nine studies) or another viral epidemic such as Zika (one study). Most of the studies show the importance of using physical and social distancing at the start of the epidemic and utilising a staggered approach when easing the restrictions, while scaling up testing. The lifting of lockdown measures should be accompanied by continued use of personal protective equipment, the limiting of workdays, and wide-scale testing.

**Conclusion:**

This review highlights the importance of timeous action when faced with an epidemic, let alone a pandemic. The setting up and relaxation of public measures are time sensitive and data-driven actions. In the absence of a safe and effective vaccine, these findings are relevant for the sustainable containment of COVID-19 in African countries.

## Introduction

Physical and social distancing are used to keep a safe distance between people, whether they are infected or not, to limit coronavirus transmission [1-3]. In South Africa, for example, these public health measures started with school closures, prohibition of gatherings of more than 100 people, and ban on travel from countries with community transmission of coronavirus [2]. As the number of cases continued to rise, the country instituted a restrictive national lockdown requiring South Africans to stay at home and only go outside their homes for essential services such as buying food and medicine. However, although physical and social distancing may alter an entire epidemic´s dynamics, these measures are not sustainable over a long period [1]. They can have negative effects on social structures as well as on the economy. Thus, after the first wave of the epidemic, plans on relaxing the measures should begin [1]. This paper seeks to examine the evidence on the setting up and relaxation of public health measures for containing coronavirus disease 2019 (COVID-19).

## Methods

We conducted searches in PubMed, Scopus, and Google Scholar [4]; with no restrictions or limiters. In our search strategy, we used combinations of the following keywords: *relaxed, measures, restrictions, exit strategy,* and COVID-19. The search strategy for PubMed is shown in Table 1. One researcher (JR) conducted the searches, screened the search output, assessed study eligibility, and extracted data; discussing each step with a second researcher (CSW). One researcher (JR) extracted data and translated these into a logical and descriptive summary of the findings.

**Table 1:** PubMed search strategy

	Search string
#1	(relaxed restrictions OR relaxed measures) AND COVID-19
#2	COVID-19 AND (exit strategy OR relaxed measures OR restrictions OR setting up OR distancing)
#3	#1 OR #2

## Results

**Figure 1:**
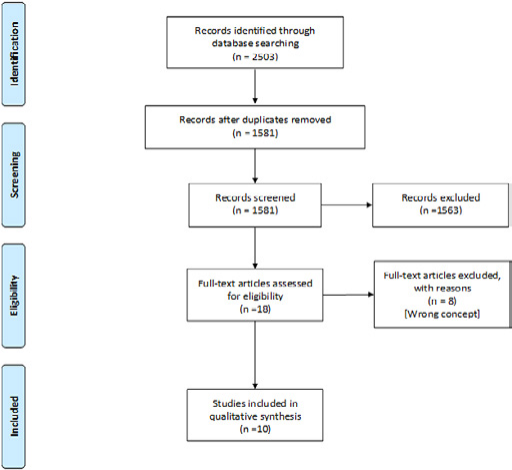
Flow diagram showing the search and selection of studies for the review

The electronic searches yielded 2503 hits, from which we included 10 eligible articles [5-14]. Figure 1 shows the search and selection process for the review. We have summarised the characteristics and findings of the 10 included studies in Tables 2 and 3. Five studies used mathematical modeling or regression trend analysis to assess when to set up and eventually lift physical and social distancing restrictions [5,6,9,10,13]. These studies used COVID-19 data from Wuhan China [5,6], their own country or multiple countries [12], or data from past epidemics of another viral disease [11]. Most of the studies spoke to using immediate lockdown at the start of the epidemic, followed by a phased or staggered approach in easing restrictions whilst using wide-scale testing for localized quarantine and maintaining physical and social distancing for at-risk populations such as the elderly. The studies using mathematical models proposed using such models to predict the best times to lower restrictions. The main model used was either the susceptible-exposed-infected-recovered (SEIR) or susceptible-infected-recovered (SIR) [5,6,13]. The studies suggest that when restrictions are lowered, personal protective equipment should continue to be used [7], workdays should be limited to restrict prolonged exposure [10], and wide-scale testing should be initiated and sustained [8,11-14]. The findings of the other five observational studies [7,8,11,12,14] are consistent with the findings of the five mathematical modeling studies [5,6,9,10,13].

**Table 2:** Characteristics and summary of findings of included mathematical modeling studies

ID	Setting	Study design	Description of measures	Summary of findings	
				Setting up restrictions	Relaxing restrictions
Prem 2020 [5]	Wuhan, China	Mathematical modeling	Used an age-structured susceptible-exposed-infected-removed (SEIR) model for several physical distancing measures. They divided the population according to the infection status into susceptible (S), exposed (E), infected (I), and removed (R) individuals, and according to age into 5-year bands until age 70 years and a single category aged 75 and older (resulting in 16 age categories).	For this information one needed to know how fast COVID-19 spread through the population during the early stages of the outbreak. This was noted as difficult to determine as the true number of cases that can transmit infection at a given time is unknown and varies over time. But when this information is somewhat known, transmission dynamics will determine specifics about instituting timeous non-pharmaceutical interventions like physical distancing.	They then simulated lifting the control measures by allowing people to return to work in a phased manner and examined the effects of returning to work at different stages of the underlying outbreak (at the beginning of March or April). Findings showed restrictions maintained until April, would help to delay the epidemic peak and premature, sudden lifting of interventions could result in an earlier secondary peak, which could be flattened by relaxing the interventions gradually through a staggered approach.
Colbourn, 2020 [6]	China, South Korea, UK	Commentary	Commentary on the Prem 2020 study described in table 2	Incorporate testing, contact tracing, and localised quarantine of suspected cases as the alternative intervention strategy to distancing lockdown measures, either at the start of the epidemic (if small)	Incorporate localized quarantine of suspected cases after relaxed quarantine.
Pérez-Garcıa 2020 [9]	China, Italy and Spain	Mathematical modeling	Differential equation Meta population model (described as an “age-structured model accounting for the short-term (i.e. months) dynamics of the COVID-19 disease at the population level” pg 2)	Study showed that the first cases appeared around 25 January 2020 with quarantine implemented in March. According to the article this action had a substantial effect on decreasing infection spread.	The study proposed a weekly cycle of 2 work days and 5 lockdown days or similar strategy, such as a cycle of 4 work days and 10 lockdown days, to keep infection low. This includes those 60 years and above stay in continuous quarantine. This arrangement might sustain the economy but not without huge losses as more than half of the labour days are lost.
Stedman 2020 [10]	UK	Polynomial trend analysis	Predictions made on regression analysis of COVID-19 and community characteristics.	When UK discovered their first cases 29 January 2020, March 17th the government instituted a ‘track and containment’ and ‘migration’ policy, followed by initiating social distancing on 23rd March.	The study concludes understanding the historic recovery in the community, is vital for policy makers to determine when to relax restrictions.
Friedman 2020 [13]	US	Mathematical modeling	Stochastic Susceptible-Infected-Removed (SIR) network model	To divide population into groups (“zones”), allowing interactions within a group than interactions between groups. If a zone (group of people) becomes infected, it can be completely isolated from other zones (pg. 2). Example, older people can social in small groups doing senior activities.	Zone-based social distancing can also be a way to relax wide-scale lockdown.

**Table 3:** Characteristics and summary of findings of observational studies included in the review

ID	Setting	Study design	Description of measures	Summary of findings	
				Setting up restrictions	Relaxing restrictions
Dullien 2020 [7]	Germany	Situation analysis	Evaluation of current pandemic data in identifying factors which would influence Germany’s exit strategy from COVID-19 lockdown.	Begin lockdown with schools and workplaces.	Restrictions if to be relaxed, should be done gradually, open areas like schools can still make use of PPE's and plexiglass to limit contagion risk, and then to use resources for tracking infection chains (immigrant workers)
Peto 2020 [8]	UK	Correspondence	Suggesting weekly antigen testing to determine lockdown measures in terms of selective quarantine and in resuming economic activities.	The study recommends weekly evaluation of severe acute respiratory syndrome coronavirus 2 (SARS-CoV-2) antigen testing of the whole population in an entire city as a demonstration site (preferably several towns and cities, if possible), implementing strict household quarantine after a positive test.	Quarantine would end when all residents of the household test negative at the same time; everyone else in the city can resume normal daily activities.
Lawton 2020 [11]	UK	Commentary	Expert opinion from epidemiologists.	First part of strategy – The **hold** strategy entails lockdown until the rate of new infections falls close to zero, then lifts the lockdown and pivot to an aggressive containment strategy.	The **hold** strategy entails lockdown until the rate of new infections falls close to zero, then lifts the lockdown and pivot to an aggressive containment strategy. The author describes this as diagnosing second-wave cases as quickly as possible, isolating them, tracing their contacts and isolating them too, if required to cut all new lines of transmission. The **build** strategy notes buying time to capacitate health services for second wave of infection whilst recovering from the first. Lastly, **shield** would mean to end lockdown but protect the most vulnerable from the virus-would require widespread screening.
Kupferschmidt 2020 [12]	US, UK, Netherlands, Singapore, Hong Kong, and South Korea	Commentary	Situation analysis of global COVID-19 data from countries with highest COVID-19 rates.	Singapore, Hong Kong, and South Korea identified and quarantined cases whilst using contact tracing who were also placed in quarantine, while imposing light restrictions on the rest of society. Strategy will depend on wide-scale testing, which is challenged by the lack of reagents and materials.	Easing social distancing measures, then reintroduce restrictions when infections begin to peak. A strategy Singapore and Hong Kong are both pursuing.
WHO 2020 [14]	Global	Interim guidelines	Review	Setting up of measures based on risk assessment using scientific findings and real-world experience.	Lifting measures in a controlled, stepwise manner (gradual re-entry into workplace) but also carrying out wide-scale testing.

## Discussion

The findings of this paper suggest timely initiation of social and physical distancing measures to limit the spread of virus transmission, followed by a phased approach when relaxing these public health measures. This process should be iterative, dependent on the rise and fall of infection rates during the pandemic duration. Half of the studies in this paper used mathematical predictions to aid decision-making regarding physical and social distancing for containing the COVID-19 pandemic. Ivorra and colleagues note that modelling is a vital tool for decision-making to control human and animal disease spread, but they caution that for real world impact one must create case specific models due to biological characteristics unique to each disease [15]. The ‘suppress and lift’ strategy, addressed by four studies in this paper [5,7,11,12], consists of instituting strict measures during infection peak and lifting the restrictions when infection rates decrease to an acceptable range [16]. The action of instituting restriction measures and lifting them will be intermittent during the epidemic period [16]. It is predicted that a once-off period of social distancing interventions may not be enough, but that a series of sporadic social distancing measures may be needed well into 2022; with the need for surveillance and monitoring of possible contagion resurgence occurring until 2024 [17]. Some level of social distancing may have to be maintained once restrictions are lifted; however, in a manner where such measures are targeted instead of general [16]. In addition, continuous surveillance must take place and further decisions based on real-time data, as it is likely that COVID-19 may evolve in seasonal patterns such as those seen with influenza [16].

The studies in this paper are mostly focused on the global North, perhaps due to their high COVID-19 rates. However, African countries may be negatively impacted by COVID-19 containment measures, due to a substantial informal economic sector and weakened health systems [18,19]. Therefore, context sensitivity is important in the institution and easing of COVID-19 containment measures in African countries and must be acknowledged by African COVID-19 task teams and command centres [18,19]. Hargreaves and colleagues provide important insights from comparing two pandemics, HIV and COVID-19 [20]. They emphasize universal access to essential preventive and treatment services, and a collaborative approach in the provision of COVID-19 interventions. As much as epidemiological models can be used to predict COVID-19 dynamics, we would need a multidisciplinary and collaborative effort in the design, characterization, implementation, and evaluation of interventions [20]. The process of setting up measures should be done timeously and their relaxation must be done in a phased approach, with a staggered re-entry into daily routine such as re-opening of schools and return to economic activities. Given the importance of real time data for this process, adequate support and infrastructure should be provided for surveillance activities to occur. The World Health Organization (WHO) and the African Union should create guidelines on how to contextualise social distancing measures. Contextualised measures would allow best practice methods to limit virus transmission, while effectively managing available resources, capacitating weakened health systems, and providing relief to citizens whose lives and livelihoods have been disrupted by COVID-19 and its containment measures. Care, services and interventions during the pandemic should bridge inequality, not elevate it [20,21].

## Conclusion

The paper highlights that when faced with an epidemic let alone a pandemic, reactions need to be timeous. Whether setting up or relaxing measures all these actions are time sensitive and require up to date information for vital decision making, as seen in most of the studies included in this paper. Whichever strategy was addressed, the need for a gradual phased approach was unanimous; with emphasis placed on health system capacity and continued social distance measures to be in place for successful viral suppression. Furthermore, for efficacy to be guaranteed, we must ensure that measures are contextualised to each country setting.

### What is known about this topic

- Social and physical distancing measures limit virus transmission and delay the COVID-19 peak;
- Though effective, physical and social distancing should not be carried over an extended time due to negative socio-economic effects.

### What this study adds

- Relaxing physical and social distancing measures should be done in a phased and iterative manner;
- Decisions on physical and social distancing measures should be contextualized, using real-time country-specific data.

## Competing interests

The authors declare no competing interests.
